# Chemotherapy refractory testicular germ cell tumor is associated with a variant in Armadillo Repeat gene deleted in Velco-Cardio-Facial syndrome (*ARVCF*)

**DOI:** 10.3389/fendo.2012.00163

**Published:** 2012-12-13

**Authors:** Chunkit Fung, David J. Vaughn, Nandita Mitra, Stephanie L. Ciosek, Saran Vardhanabhuti, Katherine L. Nathanson, Peter A. Kanetsky

**Affiliations:** ^1^Department of Hematology and Oncology, Wilmot Cancer Center, University of Rochester Medical CenterRochester, NY, USA; ^2^Department of Hematology and Oncology, Abramson Cancer Center, University of PennsylvaniaPhiladelphia, PA, USA; ^3^Department of Biostatistics and Epidemiology, Perelman School of Medicine, University of PennsylvaniaPhiladelphia, PA, USA; ^4^Division of Translational Medicine and Human Genetics, Department of Medicine, Perelman School of Medicine, University of PennsylvaniaPhiladelphia, PA, USA; ^5^Center for Biostatistics in AIDS research, Harvard School of Public HealthBoston, MA, USA

**Keywords:** *GSTP1*, *COMT*, *TPMT*, *ARVCF*, testicular germ cell tumor, refractory disease, neuropathy, ototoxicity

## Abstract

**Introduction:** There is evidence that inherited genetic variation affects both testicular germ cell tumor (TGCT) treatment outcome and risks of late-complications arising from cisplatin-based chemotherapy. Using a candidate gene approach, we examined associations of three genes involved in the cisplatin metabolism pathway, *GSTP1*, *COMT*, and *TPMT*, with TGCT outcome and cisplatin-induced neurotoxicity. **Materials and Methods:** Our study population includes a subset of patients (*n* = 137) from a genome-wide association study at the University of Pennsylvania that evaluates inherited genetic susceptibility to TGCT. All patients in our study had at least one course of cisplatin-based chemotherapy with at least 1 year of follow-up. A total of 90 markers in *GSTP1*, *COMT*, and *TPMT* and their adjacent genomic regions (±20 kb) were analyzed for associations with refractory TGCT after first course of chemotherapy, progression-free survival (PFS), overall survival (OS), peripheral neuropathy, and ototoxicity. **Results:** After adjustment for multiple comparisons, one Single nucleotide polymorphism (SNP), rs2073743, in the flanking region (±20 kb) of *COMT* was associated with refractory TGCT after initial chemotherapy. This SNP lies within the intron region of the Armadillo Repeat gene deleted in Velco-Cardio-Facial syndrome (*ARVCF*). The G allele of rs2073743 predisposed patients to refractory disease with a relative risk of 2.6 (95% CI 1.1, 6.3; *P* = 0.03). Assuming recessive inheritance, patients with the GG genotype had 22.7 times higher risk (95% CI 3.3, 155.8; *P* = 0.04) of developing refractory disease when compared to those with the GC or CC genotypes. We found no association of our candidate genes with peripheral neuropathy, ototoxicity, PFS and OS. **Discussion:** This is the first study to suggest that germline genetic variants of *ARVCF* may affect TGCT outcome. The result of this study is hypothesis generating and should be validated in future studies.

## Introduction

Testicular germ cell tumor (TGCT) is the most common solid malignancy that affects men between the ages 15 and 35 (National Cancer Institute, 2011). Cisplatin-based chemotherapy is the standard treatment for patients with metastatic disease and is highly effective with a 5-year survival rate that approaches 95% (Jemal et al., 2008). Since its introduction in the 1970s, there is a large and growing population of long-term survivors with TGCT. However, cisplatin-based chemotherapy causes peripheral neuropathy and ototoxicity in approximately 20–30% of long-term TGCT survivors and risk of these adverse events increases with higher cumulative dose of cisplatin (Fung and Vaughn, 2011).

Germline genetic variation affects both TGCT outcome and late-complications arising from cisplatin-based treatment. Single nucleotide polymorphisms (SNPs) of the bleomycin hydrolase (de Haas et al., 2008) (*BLMH*) and the high plasminogen-activator inhibitor 1 (de Haas et al., 2010) (*PAI-1*) genes have been shown to impact survival among TGCT patients (de Haas et al., 2008, 2010). Variants in glutathione *S*-transferase pi (*GSTP1*), which encodes an enzyme that detoxifies chemotherapeutic agents by conjugating reactive electrophiles to glutathione (Mannervik et al., 1985), appear to be important in the development of long-term peripheral neuropathy and ototoxicity in adult TGCT patients (Oldenburg et al., 2007a,b). Similarly, SNPs of thiopurine *S*-methyltransferase (*TPMT*) and catechol *O*-methyltransferase (*COMT*), which encodes enzymes that metabolize thiopurine drugs (Weinshilboum, 2006) and catecholamine containing chemical via methylation (Weinshilboum, 2006), respectively, predispose the pediatric population to increased risk of hearing loss (Ross et al., 2009) after cisplatin therapy.

In an attempt to validate and extend previous findings regarding effects of germline genetic variation on cancer outcome and treatment-related toxicities via their effects on drug metabolism (Coate et al., 2010), we used a candidate gene approach to investigate the associations of *GSTP1*, *COMT*, and *TPMT*, three genes involved in the cisplatin metabolism pathway, with treatment outcome and cisplatin-induced neurotoxicity. We hypothesized that genetic variants in *GSTP1*, *COMT*, and *TPMT* that confer higher intracellular concentration of cisplatin or its metabolites are associated with lower rates of TGCT recurrence and higher incidence of cisplatin-related neurotoxicity. To test this hypothesis, we evaluated a selected panel of SNPs in *GSTP1*, *COMT*, and *TPMT* with cancer outcome, ototoxicity, and peripheral neuropathy in an existing cohort of men with TGCT who have received at least one course of cisplatin-based chemotherapy.

## Materials and methods

### Study population

Our study cohort is derived from a subset of TGCT case subjects enrolled into an ongoing case-control study designed to evaluate inherited genetic susceptibility to TGCT. Details of the parent case-control study have been described previously (Kanetsky et al., 2009, 2011) In brief, case subjects were recruited from a network of hospitals, including the Hospital of the University of Pennsylvania (HUP), University of Pennsylvania Cancer Network, Fox Chase Cancer Center, and also from the Pennsylvania and New Jersey State Cancer Registries. Only men between ages 18 and 50 years with pathologically confirmed TGCT were included, and those with human immunodeficiency virus, Klinefelter's or Down syndrome were excluded. All enrolled patients with TGCT who underwent cisplatin chemotherapy at HUP were potentially eligible for the current study. A total of 137 patients had at least one course of cisplatin-based chemotherapy, at least 1 year of follow-up since initiation of cisplatin-based chemotherapy and at least one clinical consultation with an oncologist at HUP and are included in the final analytic cohort.

The study was approved by the institutional review board at the University of Pennsylvania. All patients gave written informed consent.

### Genotyping for SNPs of *GSTP1, COMT*, and *TPMT*

All study participants had previously been genotyped using the Affymetrix Genome-Wide Human SNP Array 6.0 platform; details of genotyping efforts and quality control measures have previously been reported (Kanetsky et al., 2009, 2011). A total of 90 SNP markers with genotype call rates ≥ 90% that mapped to the genomic regions (±20 kb) of *COMT, TPMT*, and *GSTP1* (43, 44, and 3 markers, respectively) were used for analysis. A complete listing of annotated SNP markers are available from the authors by request.

### Treatment response assessment

A complete review of medical records was performed to abstract information regarding treatment response, patient demographics, tumor characteristics, and treatment information. Monitoring for treatment response included both serologic [alpha fetal protein (AFP) and beta human chorionic gonadotropin (β-HCG)] and radiographic studies (plain radiography, computed tomography, or magnetic resonance imaging).

We evaluated three treatment efficacy endpoints: (1) refractory disease after first course of chemotherapy, (2) progression-free survival (PFS), and (3) overall survival (OS). Clinical remission was defined as disappearance of all tumors by radiographic methods and normalization of both AFP and β-HCG level for at least 4 weeks after completion of first course of chemotherapy. Cancer recurrence was defined as initial clinical remission with subsequent development of disease evidenced by radiographic imaging or serology. Refractory disease was defined as persistent radiographic or serologic evidence of disease after initial course of chemotherapy.

### Neuro- and oto-toxicity assessment

We collected information regarding cisplatin-related peripheral neuropathy and ototoxicity from patients using a self-administered mailed questionnaire instrument. We adapted 12 of the 15 questions from the Michigan Neuropathy Screening Instrument (MNSI) (Moghtaderi et al., 2006) for assessment of peripheral neuropathy. For ototoxicity, we used 10 questions from an existing screening survey of hearing problem at the National Institute on Deafness and other Communications Disorders (Fung and Vaughn, 2011) with inclusion of one additional question about the presence of tinnitus.

In addition to mailing the questionnaire to all 137 patients in our study cohort, we distributed it on site to patients (*n* = 31) who had routine clinic visits at the Hospital of the University of Pennsylvania from July 2010 to June 2011. To improve the response rate, we sent reminder letters to non-responders 8 weeks after initial mailing of the questionnaire. At 12 weeks, phone calls were made to all remaining non-responders.

Patients were defined as having peripheral neuropathy if their MNSI score was two or greater. The threshold was based on the results of a MNSI validation study in subjects with diabetic peripheral neuropathy (Feldman et al., 1994). Subjects were defined as having ototoxicity if they answered “yes” to three or more of the 11 questions.

### Statistical analysis

We summarized the baseline characteristics of our population using means and standard deviations for continuous variables (age at diagnosis, age at initiation of chemotherapy, initial cisplatin dose, and cumulative life-time cisplatin dose) and proportions for categorical variables [genetically inferred race from GWAS, histology, International Germ Cell Consensus Classification (IGCCC), primary site of tumor, and type of chemotherapy]. We compared the baseline characteristics between cohorts with and without completed toxicity questionnaires using Fisher's exact test for categorical variables and *t*-test for continuous variables. We used logistic regression models to investigate associations between baseline characteristics and peripheral neuropathy, ototoxicity, and refractory disease after first course of chemotherapy. We conducted a time to event analysis using a Cox proportional hazards model to assess the effects of baseline covariates on PFS and OS.

We summarized the major and minor allele frequencies of each SNP marker and determined if their genotypic distributions were in Hardy–Weinberg equilibrium.

We studied associations of SNP markers with endpoints assuming co-dominant, recessive, and dominant genetic models using Pearson's chi-squared tests. We accounted for multiple comparisons by adjusting the *p*-values of these associations using the method of Benjamini and Hochberg (1995). We used multivariable logistic regression models to assess associations of genetic markers with peripheral neuropathy and ototoxicity, after adjusting for race, IGCCC prognostic group, primary site of disease, type of chemotherapy, and cumulative dose of cisplatin. To assess the association between each SNP and refractory disease after initial chemotherapy, we used multivariable logistic regression models with adjustment for race, IGCCC prognostic group, primary site of disease, type of chemotherapy, and dose of cisplatin during first course of chemotherapy. Since refractory disease accounts for less than 10% of all treatment outcomes, estimates from the logistic regression model are interpreted and reported as relative risks.

To assess the association between each SNP and PFS or OS, we used Cox proportional hazards models. In these time-to-event models, patients contributed to the analyses from the initiation of chemotherapy until date of last follow-up or death in the OS model with inclusion of cancer recurrence as an additional censoring event in the PFS model. In the PFS model, we adjusted for race, IGCCC prognostic group, primary site of disease, type of chemotherapy, and dose of cisplatin during first course of chemotherapy. In the OS model, we adjusted for race, IGCCC prognostic group, primary site of disease, type of chemotherapy, and cumulative dose of cisplatin. We report hazard ratios (HR) and corresponding 95% CI from the Cox models.

All statistical analyses were performed using Stata 12 (StataCorp LP, Texas, 2011).

## Results

### Baseline characteristics of patients

Baseline characteristics of all 137 patients and the subset of 66 patients (48.2%) who completed the toxicity questionnaire are given in Table 1. As expected, whites account for the majority of patients in both groups (86.8% in the entire cohort and 93.9% in the questionnaire subgroup) and the mean age at TGCT diagnosis is similar (31.0 years and 32.7 years, respectively). Tumor characteristics are similar between the groups: the predominant histology is non-seminoma (81.7% in the entire cohort and 75.8% in the questionnaire subset), the primary site of presentation is the testicle (89.0% and 90.9%, respectively), and the majority of tumors are classified as good prognosis at initiation of chemotherapy by the IGCCC criteria (68.6% and 80.3%, respectively). For both groups, bleomycin, etoposide, and cisplatin (BEP) is the most common initial chemotherapy regimen (62.0% of the entire group and 59.1% of the questionnaire subset) and the mean cumulative cisplatin dose (mg/m^2^) is comparable among them (410.9 and 404.5, respectively).

**Table 1:** **Baseline characteristics of patients**.

		**All patients (*n* = 137)**		**Patients with completed toxicity questionnaire (*n* = 66)**		**Patients without completed toxicity questionnaire (*n* = 71)**		***p*-value^a^**
**DEMOGRAPHICS**								
Race	White	119	86.8%	62	93.9%	57	80.3%	*0.02*
	Black	5	3.7%	0	0.0%	5	7.0%	
	Hispanic	4	2.9%	0	0.0%	4	5.7%	
	Asian	5	3.7%	3	4.6%	2	2.8%	
	Other	4	2.9%	1	1.5%	3	4.2%	
Mean age at diagnosis ± SD (years)		31.0 ± 8.5		32.7 ± 8.6		29.5 ± 8.3		*0.03*
**CHARACTERISTICS OF TGCT**								
Histology	Seminoma	25	18.3%	16	24.2%	9	12.7%	0.12
	Non-seminoma	112	81.7%	50	75.8%	62	87.3%	
Primary site at presentation	Testicle	122	89.0%	60	90.9%	62	87.3%	0.24
	Retroperitoneum	6	4.4%	4	6.1%	2	2.8%	
	Mediastinum	9	6.6%	2	3.0%	7	9.9%	
IGCCC prognosis group^b^	Good	94	68.6%	53	80.3%	41	57.7%	*0.01*
	Intermediate	11	8.0%	5	7.6%	6	8.5%	
	Poor	31	22.6%	8	12.1%	23	32.4%	
	Unknown	1	0.7%	0	0.0%	1	1.4%	
**CHARACTERISTICS OF CHEMOTHERAPY**								
Type of initial chemotherapy	EP	46	33.6%	26	39.4%	20	28.2%	0.35
	BEP	85	62.0%	39	59.1%	46	64.8%	
	TIP	1	0.7%	0	0.0%	1	1.4%	
	VIP	4	2.9%	1	1.5%	3	4.2%	
	Other	1	0.7%	0	0.0%	1	1.4%	
Mean initial total cisplatin dose ± SD (mg/m^2^)^c^		345.3 ± 80.4		337.9 ± 80.0		352.1 ± 82.6		0.30
Mean cumulative cisplatin dose ± SD (mg/m^2^)^d^		410.9 ± 169.2		404.5 ± 166.8		416.9 ± 172.4		0.67
Mean age at initiation of chemotherapy ± SD (year)		31.5 ± 9.1		33.4 ± 9.3		29.7 ± 8.5		*0.02*

*SD, standard deviation; TGCT, testicular germ cell tumor; EP, etoposide and cisplatin; BEP, bleomycin, etoposide, and cisplatin; TIP, paclitaxel, ifosamide, and cisplatin; VIP, etoposide, ifosamide, and cisplatin*.

a *P-values for comparison of baseline characteristics between cohorts with and without completed toxicity questionnaires. Fisher's exact test used for categorical variables and t-test used for continuous variables*.

b *IGCCC prognosis group of TGCT at initiation of first course of chemotherapy*.

c *Mean total cisplatin dose administered during first course of chemotherapy*.

d *Mean cumulative life-time cisplatin dose administered by the end of last follow-up visit*.

*Italic values indicate p ≤ 0.05*.

In general, patients who completed the toxicity questionnaire are similar to the non-responders. There are no differences in terms of histology, primary site of TGCT, types of initial chemotherapy, or cumulative cisplatin dose administered (*P* > 0.05). However, those who completed the questionnaire were older at the time of diagnosis of TGCT (*P* = 0.03) and hence at initiation of chemotherapy (*P* = 0.02). A larger proportion of non-responders were non-whites (19.7% versus 6.1%, *P* = 0.02) and also had poor risk disease (32.4% versus 12.1%, *P* = 0.01) when compared to those who completed the questionnaire.

### Peripheral neuropathy and ototoxicity

Among the 66 patients who completed the toxicity questionnaire, 25 (37.9%) and 30 (45.5%) of them reported peripheral neuropathy and ototoxicity, respectively (Table 2). Patients who were diagnosed with TGCT at an older age were more likely to report developing peripheral neuropathy (*OR* = 3.1, 95% CI 1.5, 6.4 per 10 year increase in age) as were patients with higher cumulative life-time cisplatin dose (*OR* = 1.6, 95% CI 1.1, 2.2 per 100mg/m^2^ increased in dose). Those with non-seminoma (*OR* = 0.3, 95% CI 0.1, 0.8) and BEP (*OR* = 0.3, 95% CI 0.1, 0.95) as initial treatment had statistically significantly reduced risk of peripheral neuropathy compared to those with seminoma or treatment with etoposide and cisplatin (EP), respectively. Regarding ototoxicity, only higher cumulative life-time cisplatin dose (*OR* = 1.5, 95% CI 1.1, 2.1 per 100mg/m^2^ increased in dose) and total cisplatin dose (*OR* = 2.1, 95% CI 1.03, 4.3 per 100mg/m^2^ increased in dose) during initial chemotherapy increased this risk significantly.

**Table 2:** **Risk factors for development of peripheral neuropathy and ototoxicity of 66 patients who completed the toxicity questionnaire**.

	**Peripheral neuropathy**					**Ototoxicity**				
	**Yes N (%)**	**No N (%)**	**OR**	**95% CI**	***P*-value**	**Yes N (%)**	**No N (%)**	**OR**	**95% CI**	***P*-value**
**AGE AT DIAGNOSIS^a^**										
Mean ± SD (year)	37.0 ± 8.7	30.0 ± 7.4	***3.1***	***1.5, 6.4***	***<0.01***	33.6 ± 7.9	31.9 ± 9.1	1.3	0.7, 2.3	0.44
**RACE**										
White (*n* = 62)	23 (92.0)	39 (95.1)	1.0	–	–	30 (100)	32 (88.9)	1.0	–	–
Non-white (*n* = 4)	2 (8.0)	2 (4.9)	1.7	0.2,12.9	0.61	0 (0.0)	4 (11.1)	1.0	NA	NA
**PRIMARY SITE**										
Testicle (*n* = 60)	22 (88.0)	38 (92.7)	1.0	–	–	26 (86.7)	34 (94.4)	1.0	–	–
Extra-gonadal (*n* = 6)	3 (12.0)	3 (7.3)	1.7	0.3, 9.3	0.53	4 (13.3)	2 (5.6)	2.6	0.4, 15.4	0.29
**HISTOLOGY**										
Seminoma (*n* = 16)	10 (40.0)	6 (14.6)	1.0	–	–	9 (30.0)	7 (19.4)	1.0	–	–
Non-seminoma (*n* = 50)	15 (60.0)	35 (85.4)	***0.3***	***0.1, 0.8***	***0.02***	21 (70.0)	29 (80.6)	0.6	0.2, 1.8	0.32
**IGCCC PROGNOSIS GROUP**										
Good (*n* = 53)	20 (80.0)	33 (80.5)	1.0	–	–	23 (76.7)	30 (83.3)	1.0	–	–
Intermediate/poor (*n* = 13)	5 (20.0)	8 (19.5)	1.0	0.3, 3.6	0.96	7 (23.3)	6 (16.7)	1.5	0.5, 5.2	0.50
**TYPES OF INITIAL CHEMOTHERAPY**										
EP (*n* = 26)	14 (56.0)	12 (29.3)	1.0	–	–	12 (40.0)	14 (38.9)	1.0	–	–
BEP (*n* = 39)	11 (44.0)	28 (68.3)	***0.3***	***0.1, 0.95***	***0.04***	17 (56.7)	22 (61.1)	0.9	0.3, 2.4	0.84
VIP (*n* = 1)	0 (0)	1 (2.4)	NA	NA	NA	1 (3.3)	0 (0)	NA	NA	NA
**INITIAL TOTAL CISPLATIN DOSE^b^**										
Mean ± SD (mg/m^2^)	360.0 ± 81.6	324.4 ± 73.4	2.0	0.9, 4.1	0.08	360.0 ± 56.3	319.4 ± 88.9	***2.1***	***1.03, 4.3***	***0.04***
**CUMULATIVE CISPLATIN DOSE^c^**										
Mean ± SD (mg/m^2^)	476.0 ± 173.9	361.0 ± 148.1	***1.6***	***1.1, 2.2***	***0.01***	460.0 ± 188.6	358.3 ± 131.7	***1.5***	***1.1, 2.1***	***0.02***

*OR, odds ratio; CI, confidence interval; SD, standard deviation; NA, not applicable; EP, etoposide and cisplatin; BEP, bleomycin, etoposide, and cisplatin; VIP, etoposide, ifosamide, and cisplatin*.

a *Odds ratio per increase of 10 years of age*.

b *Odds ratio per increase of 100 mg/m^2^ of total cisplatin dose administered during first course of chemotherapy*.

c *Odds ratio per increase of 100 mg/m^2^ of cumulative life-time cisplatin dose administered by the end of last follow-up visit*.

*Bold-italic values indicate p ≤ 0.05*.

### Treatment outcome

In our cohort of 137 patients, 11 (8.0%) of them had refractory disease after initial course of chemotherapy and 126 (92%) of them achieved clinical remission. Among those with remission, 30 (23.8%) of them experienced disease relapse after a mean follow-up time of 46.8 months, with a total of eight deaths within the entire cohort. Table 3 shows that extra-gonadal primary (*RR* = 9.7, 95% CI 2.5, 37.3), intermediate/poor risk cancer (*RR* = 28.2, 95% CI 3.5, 228.7), and initial therapy with etoposide/ifosamide/cisplatin (VIP) (*RR* = 135.0, 95% CI 6.7, 2733.6) were statistically significantly associated with refractory disease. Patients with non-seminoma (*HR* = 3.4, 95% CI 1.03–10.9), extra-gonadal primary (*HR* = 3.5, 95% CI 1.7, 7.3), intermediate/poor risk cancer (*HR* = 4.1, 95% CI 2.2, 7.7), initial VIP (*HR* = 27.7, 95% CI 7.9, 96.7), and higher total cisplatin dose during initial chemotherapy (*HR* = 1.5, 95% CI 1.01–2.3 per 100mg/m^2^ increased in dose) all had worse PFS. Patients with extra-gonadal primary (*HR* = 5.5, 95% CI 1.3, 22.9), intermediate/poor risk cancer (*HR* = 17.7, 95% CI 2.2, 143.5), VIP as initial chemotherapy (*HR* = 255.7, 95% CI 12.2, 5367.1), and higher cumulative life-time cisplatin dose (*HR* = 1.6, 95% CI 1.2, 2.1 per 100mg/m^2^ increased in dose) had worse OS.

**Table 3:** **Association of baseline characteristics with testicular cancer treatment outcome of 137 patients in the study cohort**.

	**Refractory disease**					**Progression-free survival**				**Overall survival**			
	**Yes N (%)**	**No N (%)**	**RR**	**95% CI**	***P*-value**	**Mean PFS (mo)**	**HR**	**95% CI**	***P*-value**	**Mean OS (mo)**	**HR**	**95% CI**	***P*-value**
**AGE AT DIAGNOSIS^a^**													
Mean ± SD (year)	27.0 ± 5.3	31.4 ± 8.7	0.5	0.2, 1.2	0.11	46.8	1.0	0.7, 1.04	1.00	59.0	0.5	0.2, 1.3	0.17
**RACE**													
White (*n* = 119)	11 (100)	108 (85.7)	1.0	–	–	48.9	1.0	–	–	61.8	1.0	–	–
Non-white (*n* = 18)	0 (0.0)	18 (14.3)	1.0	NA	NA	33.2	0.5	0.2, 1.7	0.29	40.8	2.1 × 10^−7^	NA	1.00
**PRIMARY SITE**													
Testicle (*n* = 122)	6 (54.5)	116 (92.1)	1.0	–	–	48.8	1.0	–	–	56.5	1.0	–	–
Extra-gonadal (*n* = 15)	5 (45.5)	10 (7.9)	***9.7***	***2.5, 37.3***	***<0.01***	30.8	***3.5***	***1.7, 7.3***	***<0.01***	79.3	***5.5***	***1.3, 22.9***	***0.02***
**HISTOLOGY**													
Seminoma (*n* = 25)	0 (0)	25 (19.8)	1.0	–	–	57.7	1.0	–	–	59.0	1.0	–	–
Non-seminoma (*n* = 112)	11 (100)	101 (80.2)	1.0	NA	NA	44.4	***3.4***	***1.03, 10.9***	***0.04***	59.1	1.9 × 10^7^	NA	1.00
**IGCCC PROGNOSIS GROUP**													
Good (*n* = 94)	1 (9.1)	93 (73.8)	1.0	–	–	50.1	1.0	–	–	55.7	1.0	–	–
Intermediate/poor (*n* = 43)	10 (90.9)	33 (26.2)	***28.2***	***3.5, 228.7***	***<0.01***	39.6	***4.1***	***2.2, 7.7***	***<0.01***	66.5	***17.7***	***2.2, 143.5***	***0.01***
**TYPES OF INITIAL CHEMOTHERAPY**													
EP (*n* = 46)	1 (9.1)	45 (35.7)	1.0	–	–	40.5	1.0	–	–	51.9	1.0	–	–
BEP (*n* = 85)	7 (63.6)	78 (61.9)	4.0	0.5, 33.9	0.20	49.1	1.1	0.6, 2.2	0.75	62.3	2.7	0.3, 22.7	0.38
TIP (*n* = 1)	0 (0)	1 (0.8)	NA	NA	NA	54.2	NA	NA	NA	54.2	NA	NA	NA
VIP (*n* = 4)	3 (27.3)	1 (0.8)	***135.0***	***6.7, 2733.6***	***<0.01***	2.9	***27.7***	***7.9, 96.7***	***<0.01***	10.7	***255.7***	***12.2, 5367.1***	***<0.01***
Others (*n* = 1)	0 (0)	1 (0.8)	NA	NA	NA	315.4	NA	NA	NA	315.4	NA	NA	NA
**INITIAL TOTAL CISPLATIN DOSE^b^**													
Mean ± SD (mg/m^2^)	345.5 ± 104	345.2 ± 78.6	1.0	0.9, 1.08	1.00	46.8	***1.5***	***1.01, 2.3***	***0.04***	59.0	2.2	0.9, 5.6	0.10
**CUMULATIVE CISPLATIN DOSE^c^**													
Mean ± SD (mg/m^2^)											***1.6***	***1.2, 2.1***	***<0.01***

*RR, relative risk; CI, confidence interval; HR, hazard ratio; NA, not applicable; SD, standard deviation; EP, etoposide and cisplatin; BEP, bleomycin, etoposide, and cisplatin; TIP, paclitaxel, ifosamide, and cisplatin; VIP, etoposide, ifosamide, and cisplatin*.

a *Refractory disease: relative risk per unit increase of 10 years of age; PFS and OS: hazard ratio per unit increase of 10 years of age*.

b *Refractory disease: relative risk per unit increase of 100 mg/m^2^ of total cisplatin dose administered during first course of chemotherapy; PFS and OS: hazard ratio per unit increase of 100 mg/m^2^ of total cisplatin dose administered during first course of chemotherapy*.

c *Refractory disease and PFS: life-time cumulative cisplatin dose is not applicable since status for refractory disease or cancer relapse was ascertained after initial course of chemotherapy only; OS: hazard ratio per unit increase of 100 mg/m^2^ of cumulative life-time cisplatin dose*.

*Bold-italic values indicate p ≤ 0.05*.

### Associations of variants in *COMT, TPMT*, and *GSTP1* with peripheral neuropathy, ototoxicity, and treatment outcomes

We found statistically significant associations of four SNP markers (rs4646316, rs4380755, rs5008499, and rs6591256) in or near *COMT*, *TPMT*, and *GSTP1* with neuropathy and five SNP markers (rs3788306, rs12189790, rs17420046, rs6938294, and rs6912842) with ototoxicity at the nominal *p*-value of 0.05. However, after correcting for multiple testing, these associations did not retain statistical significance (Table 4).

**Table 4:** **Association of genetic variants that reached a nominal *p*-value of 0.05 with peripheral neuropathy and ototoxicity among 66 patients who completed the toxicity questionnaire**.

	**Gene (SNP)**	**Genotype**	**Case N (%)**	**Control N (%)**	**Univariate model**		**Multivariate model^a^**	
					**Odds ratio (95% CI)**	***p*-value**	**Odds ratio (95% CI)**	***p*-value**
Peripheral neuropathy
	**COMT**							
	rs4646316	Co-dominant genetic model						
		T/T	0 (0)	3 (7.3)	0.1 (0.02, 0.8)	0.03	0.1 (0.01, 0.7)	0.03
		T/C	9 (37.5)	23 (56.1)	0.3 (0.1, 0.9)	0.03	0.3 (0.1, 0.9)	0.03
		C/C	15 (62.5)	15 (36.6)	1.0	–	1.0	–
		Dominant genetic model						
		T/-	9 (37.5)	26 (63.4)	0.4 (0.1, 0.98)	0.05	0.3 (0.1, 0.98)	0.05
		C/C	15 (62.5)	15 (36.6)	1.0	–	1.0	–
	**TPMT**							
	rs4380755	Co-dominant genetic model						
		T/T	1 (4.3)	0 (0)	8.7 (1.02, 74.1)	0.05	18.9 (1.1, 323.8)	0.04
		T/C	9 (39.1)	8 (21.1)	2.9 (1.01, 8.6)	0.05	4.3 (1.1, 18.0)	0.04
		C/C	13 (56.5)	30 (78.9)	1.0	–	1.0	–
	rs5008499	Co-dominant genetic model						
		T/T	1 (4.5)	0 (0)	15.0 (1.5, 152.0)	0.02	14.4 (0.97, 212.0)	0.05
		T/C	8 (36.4)	6 (15.0)	3.9 (1.2, 12.3)	0.02	3.8 (0.99, 14.6)	0.05
		C/C	13 (59.1)	34 (85.0)	1.0	–	1.0	–
	**GSTP1**							
	rs6591256	Dominant genetic model						
		G/-	12 (52.2)	30 (76.9)	0.3 (0.1, 0.99)	0.05	0.3 (0.1, 1.1)	0.08
		A/A	11 (47.8)	9 (23.1)	1.0	–	1.0	–
Ototoxicity
	**COMT**							
	rs3788306	Dominant genetic model						
		G/-	20 (74.1)	17 (48.6)	3.0 (1.02, 9.0)	0.05	4.9 (1.3-18.8)	0.02
		A/A	7 (25.9)	18 (51.4)	1.0	–	1.0	–
	**TPMT**							
	rs12189790	Co-dominant genetic model						
		T/T	0 (0)	0 (NA)	13.8 (1.1, 90.1)	0.04	20.6 (1.5, 277.1)	0.02
		T/C	13 (43.3)	7 (19.4)	3.2 (1.1 9.5)	0.04	4.5 (1.2, 16.6)	0.02
		C/C	17 (56.7)	29 (80.6)	1.0	–	1.0	–
		Dominant genetic model						
		T/-	13 (43.3)	7 (19.4)	3.2 (1.1, 9.5)	0.04	4.5 (1.2, 16.6)	0.02
		C/C	17 (56.7)	29 (80.6)	1.00	–	1.0	–
	rs17420046	Co-dominant genetic model						
		T/T	1 (3.3)	2 (5.6)	0.1 (0.0, 0.6)	0.02	0.01 (0.0, 0.3)	0.01
		T/G	1 (3.3)	12 (33.3)	0.2 (0.1, 0.8)	0.02	0.1 (0.03, 0.6)	0.01
		G/G	28 (93.3)	22 (61.1)	1.0	–	1.0	–
		Dominant genetic model						
		T/-	2 (6.7)	14 (38.9)	0.1 (0.02, 0.6)	0.01	0.1 (0.01, 0.4)	0.01
		G/G	28 (93.3)	22 (61.1)	1.0	–	1.0	–
	rs6938294	Co-dominant genetic model						
		G/G	1 (3.3)	2 (5.7)	0.04 (0.0, 0.6)	0.02	0.01 (0.0, 0.3)	0.01
		G/A	1 (3.3)	12 (34.3)	0.2 (0.1, 0.8)	0.02	0.1 (0.03, 0.6)	0.01
		A/A	28 (93.3)	21 (60.0)	1.0	–	1.0	–
		Dominant genetic model						
		G/-	2 (6.7)	14 (40.0)	0.1 (0.02, 0.5)	0.01	0.04 (0.01, 0.4)	0.01
		A/A	28 (93.3)	21 (60.0)	1.0	–	1.0	–
	rs6912842	Dominant genetic model						
		T/-	13 (43.3)	7 (19.4)	3.2 (1.1, 9.5)	0.04	4.5 (1.2, 16.6)	0.02
		G/G	17 (56.7)	29 (80.6)	1.0	–	1.0	–

*CI, confidence interval*.

a *Adjusted for race, IGCCC, primary site of cancer, type of chemotherapy administered, and cumulative life-time cisplatin dose administered*.

Nine SNPs in or near *COMT*, *TPMT*, and *GSTP1* had a statistically significant association with refractory disease after initial chemotherapy at the nominal *p*-value of 0.05 (Table 5). After correcting for multiple testing, we found that recessive inheritance of the G allele of rs2073743 confers increased risk (*RR* = 22.7, 95% CI 3.3, 155.8) of refractory disease after first course of chemotherapy. However, this association did not retain statistical significance after adjustment for race, IGCCC, primary site of disease at diagnosis, type of initial chemotherapy, and total dose of cisplatin during first course of chemotherapy.

**Table 5:** **Association of genetic variants that reached a nominal *p*-value of 0.05 with refractory disease after initial chemotherapy among 137 patients in the study cohort**.

**Gene (SNP)**	**Genotype**	**Yes N (%)**	**No N (%)**	**Univariate model**		**Multivariate model^a^**	
				**Relative risk (95% CI)**	***p*-value**	**Relative risk (95% CI)**	***p*-value**
**COMT**							
rs2073743	Co-dominant genetic model						
	G/G	3 (27.3)	2 (1.6)	8.8 (1.2, 65.8)	0.04	4.7 (0.3, 69.4)	0.25
	G/C	3 (27.3)	48 (39.0)	3.0 (1.1, 8.1)	0.04	2.2 (0.6, 8.3)	0.25
	C/C	5 (45.4)	73 (59.3)	1.0	–	1.0	–
	Recessive genetic model						
	G/G	3 (27.3)	2 (1.6)	22.7 (3.3, 155.8)	<0.01^b^	21.8 (1.01, 470.7)	0.05^c^
	C/-	8 (72.7)	121 (98.4)	1.0	–	1.0	–
rs4646316	Co-dominant genetic model						
	T/T	0 (0)	6 (4.9)	0.01 (0.0, 0.6)	0.03	0.001 (0.0, 0.3)	0.02
	T/C	1 (9.1)	59 (48.0)	0.1 (0.01, 0.8)	0.03	0.03 (0.02, 0.5)	0.02
	C/C	10 (90.9)	58 (47.1)	1.0	–	1.0	–
	Dominant genetic model						
	T/-	1 (9.1)	65 (52.8)	0.1 (0.01, 0.7)	0.02	0.03 (0.001, 0.7)	0.02
	C/C	10 (90.9)	58 (47.2)	1.0	–	1.0	–
rs366148	Dominant genetic model						
	A/-	3 (27.3)	9 (7.3)	4.8 (1.07, 21.1)	0.04	2.6 (0.3, 21.5)	0.4
	G/G	8 (72.7)	114 (92.7)	1.0	–	1.0	–
rs2531706	Dominant genetic model						
	G/-	5 (45.5)	91 (76.5)	0.3 (0.1, 0.9)	0.03	0.2 (0.02, 0.97)	0.05
	A/A	6 (54.5)	28 (23.5)	1.0	–	1.0	–
rs5748505	Dominant genetic model						
	T/-	4 (36.4)	88 (69.8)	0.3 (0.1, 0.9)	0.03	0.2 (0.05, 1.2)	0.09
	C/C	7 (63.6)	38 (30.2)	1.0	–	1.0	–
rs1034564	Recessive genetic model						
	A/A	3 (30.0)	7 (5.8)	7.0 (1.5 33.0)	0.01	4.7 (0.5, 42.8)	0.2
	G/-	7 (70.0)	114 (94.2)	1.0	–	1.0	–
**TPMT**							
rs12524744	Co-dominant genetic model						
	T/T	0 (0)	0 (0)	20.6 (1.3, 319.3)	0.03	20.1 (0.5, 822.4)	0.10
	T/C	4 (40.0)	16 (12.8)	4.5 (1.2, 17.9)	0.03	4.5 (0.7, 28.7)	0.10
	C/C	6 (60.0)	109 (87.2)	1.0	–	1.0	–
	Dominant genetic model						
	T/-	4 (40.0)	16 (12.8)	4.5 (1.2, 17.9)	0.03	4.5 (0.7, 28.7)	0.10
	C/C	6 (60.0)	109 (87.2)	1.0	–	1.0	–
rs9396834	Co-dominant genetic model						
	C/C	5 (45.4)	15 (11.9)	6.6 (1.1, 40.8)	0.04	5.0 (0.6, 45.0)	0.15
	C/T	3 (27.3)	62 (49.2)	2.6 (1.03, 6.4)	0.04	2.2 (0.7, 6.7)	0.15
	T/T	3 (27.3)	49 (38.9)	1.0	–	1.0	–
	Recessive genetic model						
	C/C	5 (45.5)	15 (11.9)	6.2 (1.7, 22.71)	0.01	4.4 (0.7, 26.1)	0.10
	T/-	6 (54.5)	111 (88.1)	1.0	–	1.0	–
**GSTP1**							
rs6591256	Co-dominant genetic model						
	G/G	0 (0)	10 (8.7)	0.1 (0.01, 0.9)	0.05	0.04 (0.01, 0.93)	0.05
	G/A	4 (36.4)	67 (58.3)	0.3 (0.1, 0.97)	0.05	0.2 (0.04, 0.96)	0.05
	A/A	7 (63.6)	38 (33.0)	1.0	–	1.0	–

*CI, confidence interval*.

a *Adjusted for race, IGCCC, primary site of disease at diagnosis, type of chemotherapy administered, and total dose of cisplatin received during initial chemotherapy*.

b *Corrected p-value for multiple testing: 0.04*.

c *Corrected p-value for multiple testing: 0.72*.

The G allele of rs2073743 had a frequency of 22.76% in the cohort and its genotypic distribution was in Hardy–Weinberg proportions (*P* = 0.46). This allele was present in 40.91% of patients with refractory disease compared to 21.14% of those with clinical remission after initial chemotherapy, predisposing patients to refractory disease with a relative risk of 2.6 (95% CI of 1.1, 6.3; *P* = 0.03).

We found statistically significant association of six SNPs (rs1042781, rs2073743, rs2531706, rs5748505, rs6518598, and rs4380755) in or near *COMT*, *TPMT*, and *GSTP1* with PFS and six SNPs (rs366148, rs2239395, rs1858770, rs12158214, rs887200, and rs372534) with OS at the nominal *p*-value of 0.05 (Table 6). In particular, patients with the recessive GG genotype of rs2073743 had an inferior PFS with a HR of 3.6 (95% CI 1.1, 11.7) when compared to those with the GC or CC genotypes. However, after correcting the *p*-values for multiple testing, none of these associations remained statistically significant.

**Table 6:** **Association of genetic variants that reached a nominal *p*-value of 0.05 with progression-free and overall survival among 137 patients in the study cohort**.

	**Gene (SNP)**	**Genotype**	**Mean PFS/OS (mo)**	**Univariate model**		**Multivariate model^a^**	
				**Hazard ratio (95% CI)**	***p*-value**	**Hazard ratio (95% CI)**	***p*-value**
Progression-free survival
	**COMT**						
	rs10427871	Co-dominant genetic model					
		G/G	NA	45.7 (2.5, 845.7)	0.01	9469.3 (62.7, 1429366.1)	<0.01
		G/A	7.7	6.8 (1.6, 29.1)	0.01	97.3 (7.9, 1195.6)	<0.01
		A/A	48.4	1.0	–	1.0	–
		Dominant genetic model					
		G/-	7.7	6.8 (1.6, 29.1)	0.01	97.3 (7.9, 1195.6)	<0.01
		A/A	48.4	1.0	–	1.0	–
	rs2073743	Recessive genetic model					
		G/G	43.9	3.6 (1.1, 11.7)	0.03	1.2 (0.3, 4.6)	0.84
		C/-	46.2	1.0	–	1.0	–
	rs2531706	Dominant genetic model					
		G/-	47.5	0.5 (0.3, 0.9)	0.03	0.4 (0.2, 0.8)	0.01
		A/A	42.6	1.0	–	1.0	–
	rs5748505	Dominant genetic model					
		T/-	51.1	0.5 (0.3, 0.95)	0.03	0.5 (0.2, 0.9)	0.02
		C/C	38.3	1.0	–	1.0	–
	rs6518598	Dominant genetic model					
		A/-	51.8	0.5 (0.3, 0.9)	0.02	0.4 (0.2, 0.8)	0.01
		C/C	38.8	1.0	–	1.0	–
	**TPMT**						
	rs4380755	Recessive genetic model					
		T/T	2.0	61.8 (5.6, 681.9)	<0.01	273.9 (16.8, 4464.8)	<0.01
		C/-	49.0	1.0	–	1.0	–
Overall survival
	**COMT**						
	rs366148	Co-dominant genetic model					
		A/A	11.2	49.1 (3.4, 716.6)	<0.01	15.9 (0.3, 880.5)	0.18
		A/G	50.0	7.0 (1.8, 26.8)	<0.01	4.0 (0.5, 29.7)	0.18
		G/G	61.2	1.0	–	1.0	–
		Dominant genetic model					
		A/-	46.7	8.3 (2.0, 34.8)	<0.01	4.0 (0.5, 29.7)	0.18
		G/G	61.2	1.0	–	1.0	–
	rs2239395	Dominant genetic model					
		G/-	51.0	7.7 (1.8, 32.3)	0.01	8.9 (1.4, 57.4)	0.02
		T/T	59.6	1.0	–	1.0	–
	rs1858770	Co-dominant genetic model					
		C/C	11.2	30.5 (1.5, 608.1)	0.03	26.7 (0.3, 2150.4)	0.14
		C/A	53.2	5.5 (1.2, 24.7)	0.03	5.2 (0.6, 46.4)	0.14
		A/A	59.8	1.0	–	1.0	–
		Dominant genetic model					
		C/-	48.5	6.6 (1.3, 32.6)	0.02	5.2 (0.6, 46.4)	0.14
		A/A	59.8	1.0	–	1.0	–
	rs12158214	Recessive genetic model					
		T/T	42.4	5.4 (1.04, 27.8)	0.05	NA	NA
		C/-	61.4	1.0	–	1.0	–
	rs887200	Dominant genetic model					
		G/-	46.9	4.1 (1.02, 16.4)	0.05	4.0 (0.8, 18.7)	0.08
		A/A	62.5	1.0	–	1.0	–
	**TPMT**						
	rs372534	Co-dominant genetic model					
		G/G	10.5	26.5 (1.8, 389.7)	0.02	4.7 (0.3, 74.3)	0.27
		G/A	54.0	5.2 (1.4, 19.7)	0.02	2.2 (0.5, 8.6)	0.27
		A/A	62.6	1.0	–	1.0	–
		Recessive genetic model					
		G/G	10.5	46.9 (4.2, 528.3)	<0.01	0.6 (0.03, 12.9)	0.73
		A/-	59.9	1.0	–	1.0	–

*PFS, progression-free survival; OS, overall survival; CI, confidence interval; NA, not applicable*.

a *PFS: hazard ratio adjusted for race, IGCCC, type of chemotherapy administered, and cumulative life-time cisplatin dose administered by the end of last follow-up visit; OS: hazard ratio adjusted for race, IGCCC, primary site of cancer, type of chemotherapy administered, and cumulative life-time cisplatin dose administered by the end of last follow-up visit*.

## Discussion

This study examined the association between three genes involved in cisplatin metabolism, *GSTP1*, *COMT*, and *TPMT*, with TGCT outcomes and cisplatin-induced ototoxicity and peripheral neuropathy. We found one SNP (rs2073743) in the flanking region (±20 kb) of *COMT* that was associated with refractory TGCT after initial chemotherapy. This SNP lies within the intron region of the Armadillo Repeat gene deleted in Velo-Cardio-Facial syndrome (*ARVCF*). This is the first study to show that germline genetic variants of *ARVCF* may affect TGCT outcome.

Although there is no functional information for SNP rs2073743, we hypothesize that it may affect disease risk by altering the expression of the *ARVCF* gene. *ARVCF* gene is a member of the p120 catenin family of proteins (Reintsch et al., 2008) and its over-expression has been shown to cause disruption of cell adhesion (Reintsch et al., 2008), which may facilitate cancer progression. A recent study by Lin et al. (2011) reported that the dominant inheritance of the minor T allele of rs5993891 of *ARVCF* is associated with a decrease in prostate cancer specific mortality (*HR* = 0.21, 95% CI 0.07, 0.61). Therefore, it is possible that SNP rs2073743 of *ARVCF* may predispose patients to higher risk of refractory TGCT by inhibition of cell adhesion, which consequently causes more aggressive tumor biology.

In contrast, SNP rs2073743 may be a marker for genetic variations in *COMT* that affect the risk of refractory TGCT by altering the level of *S*-adenosyl-L-methionine (AdoMet) and the concentration of estrogen and its metabolites. *COMT* encodes a methyltransferase that is critical for the metabolism of endogenous catecholamine containing chemicals and catechol drugs with AdoMet being its intermediary metabolite (Weinshilboum, 2006). There is some evidence to suggest that cisplatin may interact synergistically with AdoMet (Ochoa et al., 2009). Ochoa and colleagues found that administration of both AdoMet and cisplatin causes a 3.0–6.2-fold increase in frequency of renal toxicity in mice while cisplatin alone causes only moderate toxicity and administration AdoMet by itself did not result in any nephrotoxicity (Ochoa et al., 2009). Consequently, it is possible that increased activity of the *COMT* enzyme causes a decrease in the concentration of AdoMet, which may in turn lead to decreased cytotoxic activity of cisplatin.

TGCTs are thought to develop from totipotent primordial germ cells (Sinisi et al., 2003) and there is evidence to suggest that a combination of high gonadotropin level coupled with unbalanced androgen and estrogen level may be a key event for TGCT development and progression (Garolla et al., 2005). Fotsis et al. (1994) reported that 2-methoxyoestriol, which is an endogenous estrogen metabolite, inhibits angiogenesis and suppresses tumor growth in mice. Since *COMT* degrades catechol metabolites from estradiol and estrone to methylated compounds (Weinshilboum, 2006), genetic variation of *COMT* may be implicated in the development and progression of TGCT. Recently, Ferlin et al. (2010) studied 17 polymorphic markers in 11 genes involved in hormone metabolism and reported that the minor A allele of rs11205 in the 17 β-hydroxysteroid dehydrogenase type 4 gene (*HSD17B4*), which is essential for metabolism of estrone to estradiol, is associated with higher risk of TGCT (*OR* = 2.73, 95% CI 1.47, 7.06). In the same study, however, they found no association of rs4680 of *COMT* with development of TGCT (Ferlin et al., 2010).

Since the association between rs2073743 with refractory disease was no longer statistically significant after adjusting for race, IGCCC, primary site of disease at diagnosis, type of initial chemotherapy, and total dose of cisplatin during first course of chemotherapy, we examined if any of these covariates are associated with rs2073743. Interestingly, we found that patients with the G/G genotype at this marker have higher odds of having both extra-gonadal primary (*OR* = 14.63, 95% CI 2.22, 96.35) and initial chemotherapy with VIP (*OR* = 45.00, 95% CI 2.77, 730.57). However, we found no such association with race, IGCCC, and total dose of cisplatin. Therefore, patients with rs2073742 may have genetic predisposition to more aggressive TGCT at presentation. This may instead explain their increased risk of refractory disease after initial chemotherapy. On the contrary, since VIP is commonly used for treatment of extra-gonadal TGCT, it simply may be a marker for extra-gonadal disease. Finally, our failure to detect any association of rs2073743 with PFS or OS may result from inadequate statistical power due to small sample size (*n* = 137) and the relatively short mean follow-up time of 46.8 months within the context of the excellent 5-year survival rate of TGCT, which approaches 95% (Fung and Vaughn, 2011).

Our study confirms the known established prognostic factors for TGCT, including both IGCCC risk groups and primary site of disease presentation, thus suggesting that our results are internally valid. Based on the original study (*n* = 5862) that validated the IGCCC (Wanderas et al., 2006), the 5-year OS for good, intermediate, and poor risk metastatic GCT are 91%, 79% and 48%, respectively while the 5-year PFS for these respective prognostic groups are 89%, 75%, and 41%. Our study showed slightly better 5-year OS for all risk groups (good risk: 98.7%, intermediate risk: 87.5%, and poor risk: 76.1%) but comparable 5-year PFS except for that of the intermediate risk group (good risk: 80.0%, intermediate risk: 21.8%, and poor risk: 44.1%). These minor differences may be attributed to the smaller sample size of our study cohort. Similarly, our study also confirmed that patients with extra-gonadal primary have worse 5-year OS (testicular: 94.7%, retroperitoneal: 83.3%, and mediastinum 75.0%) and PFS (testicular: 73.0%, retroperitoneal: 22.2%, and mediastinum 33.3%).

Our study confirmed that increased cumulative dose of cisplatin predisposed patients to both hearing loss and peripheral neuropathy. In our study, 82% of patients who received >400 mg/m^2^ of cumulative cisplatin and 38% of those with ≤400 mg/m^2^ self-reported ototoxicity, which were higher than those previously reported by Bokemeyer et al. (1998). In their study, they showed that approximately 20% of TGCT patients treated with ≤400 mg/m^2^ of cumulative cisplatin and 50% of those who received >400 mg/m^2^ developed hearing impairment after a median follow-up of 58 months, which were assessed by pure-tone audiometry (Bokemeyer et al., 1998). These differences may be explained by the more objective method of ototoxicity assessment by Bokemeyer and colleagues.

Peripheral neuropathy occurred in 45.5% of our patients, consistent with other studies which showed that 14–43% of TGCT survivors experience persistent symptomatic peripheral neuropathy after cisplatin-based chemotherapy (Hansen et al., 1989; Aass et al., 1990; Bissett et al., 1990; Boyer et al., 1990; Bokemeyer et al., 1996; Roth et al., 1988; Petersen and Hansen, 1999; von Schlippe et al., 2001). Our study reported that the odds for peripheral neuropathy increased by 1.55 times per 100 mg/m^2^ increase in cumulative cisplatin dose, which is similar to those reported by other studies (Brydoy et al., 2009; Glendenning et al., 2010). For instance, Glendenning et al. (2010) reported that there is a 1.91 times higher odds of developing peripheral neuropathy with each 200 mg/m^2^ increase in cumulative cisplatin dose. Similarly, Brydoy et al. (2009) found that there is a higher prevalence of paresthesias following five or more cycles than following one to four cycles of cisplatin-based chemotherapy in TGCT survivors. Since peripheral neuropathy is expected to be more prevalent in an older population, it is not surprising that age was a significant variable in our regression analysis.

*GSTP1*, *COMT*, and *TPMT* have been implicated in the development of cisplatin-induced hearing impairment (Oldenburg et al., 2007a,b; Ross et al., 2009) and peripheral neuropathy (Oldenburg et al., 2007a). Ross et al. (2009) demonstrated that rs12201199 of *TPMT* (*OR* = 17.0, 95% CI 2.3, 125.9) and rs9332377 of *COMT* (*OR* = 5.5, 95% CI 1.9, 15.9) are associated with cisplatin-induced hearing loss in children. Regarding *GSTP1*, Oldenburg et al. (2007a,b) found statistically significant associations between rs1695 of this gene with peripheral neuropathy and cisplatin-induced long-term hearing impairment in TGCT survivors. The A > G polymorphism of rs1695 is a missense mutation that leads to amino acid change at codon 105 (Ile > Val) (Oldenburg et al., 2007a,b) and may affect the detoxification capability of this enzyme with chemotherapeutic agents.

In our study, however, we did not find significant associations of ototoxicity and peripheral neuropathy with any of our candidate genes. The following reasons may explain our null results. First, rs12201199 of *TPMT* and rs1695 of *GSTP1* were not included in the Affymetrix Genome-Wide Human SNP Array 6.0 platform and therefore were not examined. Although one SNP of *TPMT* in our study (rs11964408) was in linkage disequilibrium with rs12201199, its relatively weak linkage with rs12201199, with a correlation coefficient of 0.65, may explain the lack of significant association with peripheral neuropathy. Second, we may have inadequate statistical power due to the small number of patients in our neurotoxicity cohort (*n* = 66) to detect such associations. For instance, the risk for development of ototoxicity with rs9332377 of *COMT* in our study was increased with an odds ratio of 1.29 (95% CI 0.54, 3.11) similar to that of Oldenberg and colleague (Ross et al., 2009). However, this association may not have reached statistically significance (*P* = 0.57) due to the small sample size of our study. Third, the mechanism for development of cisplatin-induced ototoxicity may be different in adults than in children, and therefore genes other than *COMT* and *TPMT* may be involved. Finally, unlike the studies by Oldenburg et al. (2007b) and Ross et al. (2009), patients in our study used a questionnaire instead of audiometric testing to screen for hearing impairment. As a result, misclassification of hearing loss may explain our failure to detect any association with ototoxicity.

The association between SNP rs2073743 of *ARVCF* and refractory TGCT should first be replicated in future studies with a larger number of patients and subsequently validated using a different patient cohort. In particular, studies to elucidate the function of the *ARVCF* gene in the pathogenesis and progression of TGCT may give insight into development of therapeutic options in TGCT treatment. If indeed presence of the G/G of the *ARVCF* gene SNP rs2073743 is associated with refractory TGCT, incorporation of this prognostic factor into the IGCCC may be warranted.

In conclusion, our pilot data suggests that patients with the G/G genotype of the *ARVCF* gene SNP rs2073743 may have increased risk of refractory TGCT after chemotherapy. Although the underlying mechanism for this increased risk of refractory disease is unknown, confirmation of the observed association may have consequences for risk classifications in patients with metastatic disease and may be of use to select patients who will benefit from more aggressive treatment at its onset.

### Conflict of interest statement

The authors declare that the research was conducted in the absence of any commercial or financial relationships that could be construed as a potential conflict of interest.

## References

[B1] Aass N. Kaasa S. Lund E. Kaalhus O. Heier M. S. Fossa S. D. (1990). Long-term somatic side-effects and morbidity in testicular cancer patients. Br. J. Cancer 61, 151–155 2297486 10.1038/bjc.1990.31 PMC1971317

[B2] Benjamini Y. Hochberg Y. (1995). Controlling the false discovery rate – a practical and powerful approach to multiple testing. J. R. Stat. Soc. B 57, 289–300

[B3] Bissett D. Kunkeler L. Zwanenburg L. Paul J. Gray C. Swan I. R. (1990). Long-term sequelae of treatment for testicular germ cell tumours. Br. J. Cancer 62, 655–659 2171622 10.1038/bjc.1990.350 PMC1971502

[B4] Bokemeyer C. Berger C. C. Hartmann J. T. Kollmannsberger C. Schmoll H. J. Kuczyk M. A. (1998). Analysis of risk factors for cisplatin-induced ototoxicity in patients with testicular cancer. Br. J. Cancer 77, 1355–1362 9579846 10.1038/bjc.1998.226 PMC2150148

[B5] Bokemeyer C. Berger C. C. Kuczyk M. A. Schmoll H. J. (1996). Evaluation of long-term toxicity after chemotherapy for testicular cancer. J. Clin. Oncol. 14, 2923–2932 8918489 10.1200/JCO.1996.14.11.2923

[B6] Boyer M. Raghavan D. Harris P. J. Lietch J. Bleasel A. Walsh J. C. (1990). Lack of late toxicity in patients treated with cisplatin-containing combination chemotherapy for metastatic testicular cancer. J. Clin. Oncol. 8, 21–26 1688614 10.1200/JCO.1990.8.1.21

[B7] Brydoy M. Oldenburg J. Klepp O. Bremnes R. M. Wist E. A. Wentzel-Larsen T. (2009). Observational study of prevalence of long-term Raynaud-like phenomena and neurological side effects in testicular cancer survivors. J. Natl. Cancer Inst. 101, 1682–1695 10.1093/jnci/djp413 19940282 PMC2794301

[B8] Coate L. Cuffe S. Horgan A. Hung R. J. Christiani D. Liu G. (2010). Germline genetic variation, cancer outcome, and pharmacogenetics. J. Clin. Oncol. 28, 4029–4037 10.1200/JCO.2009.27.2336 20679599

[B9] de Haas E. C. Zwart N. Meijer C. Nuver J. Boezen H. M. Suurmeijer A. J. (2008). Variation in bleomycin hydrolase gene is associated with reduced survival after chemotherapy for testicular germ cell cancer. J. Clin. Oncol. 26, 1817–1823 10.1200/JCO.2007.14.1606 18398146

[B10] de Haas E. C. Zwart N. Meijer C. Suurmeijer A. J. Meijer K. Guchelaar H. J. (2010). Association of PAI-1 gene polymorphism with survival and chemotherapy-related vascular toxicity in testicular cancer. Cancer 116, 5628–5636 10.1002/cncr.25300 20737565

[B11] Feldman E. L. Stevens M. J. Thomas P. K. Brown M. B. Canal N. Greene D. A. (1994). A practical two-step quantitative clinical and electrophysiological assessment for the diagnosis and staging of diabetic neuropathy. Diabetes Care 17, 1281–1289 7821168 10.2337/diacare.17.11.1281

[B12] Ferlin A. Ganz F. Pengo M. Selice R. Frigo A. C. Foresta C. (2010). Association of testicular germ cell tumor with polymorphisms in estrogen receptor and steroid metabolism genes. Endocr. Relat. Cancer 17, 17–25 10.1677/ERC-09-0176 19776291

[B13] Fotsis T. Zhang Y. Pepper M. S. Adlercreutz H. Montesano R. Nawroth P. P. (1994). The endogenous oestrogen metabolite 2-methoxyoestradiol inhibits angiogenesis and suppresses tumour growth. Nature 368, 237–239 10.1038/368237a0 7511798

[B14] Fung C. Vaughn D. J. (2011). Complications associated with chemotherapy in testicular cancer management. Nat. Rev. Urol. 8, 213–222 10.1038/nrurol.2011.26 21403662

[B15] Garolla A. Ferlin A. Vinanzi C. Roverato A. Sotti G. Artibani W. (2005). Molecular analysis of the androgen receptor gene in testicular cancer. Endocr. Relat. Cancer 12, 645–655 10.1677/erc.1.00954 16172197

[B16] Glendenning J. L. Barbachano Y. Norman A. R. Dearnaley D. P. Horwich A. Huddart R. A. (2010). Long-term neurologic and peripheral vascular toxicity after chemotherapy treatment of testicular cancer. Cancer 116, 2322–2331 10.1002/cncr.24981 20225230

[B17] Hansen S. W. Helweg-Larsen S. Trojaborg W. (1989). Long-term neurotoxicity in patients treated with cisplatin, vinblastine, and bleomycin for metastatic germ cell cancer. J. Clin. Oncol. 7, 1457–1461 2476531 10.1200/JCO.1989.7.10.1457

[B18] Jemal A. Siegel R. Ward E. Hao Y. Xu J. Murray T. (2008). Cancer statistics, 2008. CA. Cancer J. Clin. 58, 71–96 10.3322/CA.2007.0010 18287387

[B19] Kanetsky P. A. Mitra N. Vardhanabhuti S. Li M. Vaughn D. J. Letrero R. (2009). Common variation in KITLG and at 5q31.3 predisposes to testicular germ cell cancer. Nat. Genet. 41, 811–815 10.1038/ng.393 19483682 PMC2865677

[B20] Kanetsky P. A. Mitra N. Vardhanabhuti S. Vaughn D. J. Li M. Ciosek S. L. (2011). A second independent locus within DMRT1 is associated with testicular germ cell tumor susceptibility. Hum. Mol. Genet. 20, 3109–3117 10.1093/hmg/ddr207 21551455 PMC3131044

[B21] Lin D. W. FitzGerald L. M. Fu R. Kwon E. M. Zheng S. L. Kolb S. (2011). Genetic variants in the LEPR, CRY1, RNASEL, IL4, and ARVCF genes are prognostic markers of prostate cancer-specific mortality. Cancer Epidemiol. Biomarkers Prev. 20, 1928–1936 10.1158/1055-9965.EPI-11-0236 21846818 PMC3169727

[B22] Mannervik B. Alin P. Guthenberg C. Jensson H. Tahir M. K. Warholm M. (1985). Identification of three classes of cytosolic glutathione transferase common to several mammalian species: correlation between structural data and enzymatic properties. Proc. Natl. Acad. Sci. U.S.A. 82, 7202–7206 3864155 10.1073/pnas.82.21.7202 PMC390817

[B23] Moghtaderi A. Bakhshipour A. Rashidi H. (2006). Validation of Michigan neuropathy screening instrument for diabetic peripheral neuropathy. Clin. Neurol. Neurosurg. 108, 477–481 10.1016/j.clineuro.2005.08.003 16150538

[B24] National Cancer Institute. (2011). SEER Cancer Statistics Review, 1975–2008. [Accessed October 12, 2011]. Available online at: http://cancercontrol.cancer.gov/ocs/prevalence/prevalence.html#male

[B25] Ochoa B. Bobadilla N. Arrellin G. Herrera L. A. (2009). S-Adenosyl-L-methionine increases serum BUN and creatinine in cisplatin-treated mice. Arch. Med. Res. 40, 54–58 10.1016/j.arcmed.2008.10.006 19064128

[B26] Oldenburg J. Kraggerud S. M. Brydoy M. Cvancarova M. Lothe R. A. Fossa S. D. (2007a). Association between long-term neuro-toxicities in testicular cancer survivors and polymorphisms in glutathione-s-transferase-P1 and -M1, a retrospective cross sectional study. J. Transl. Med. 5, 70 10.1186/1479-5876-5-70 18162130 PMC2245909

[B27] Oldenburg J. Kraggerud S. M. Cvancarova M. Lothe R. A. Fossa S. D. (2007b). Cisplatin-induced long-term hearing impairment is associated with specific glutathione s-transferase genotypes in testicular cancer survivors. J. Clin. Oncol. 25, 708–714 10.1200/JCO.2006.08.9599 17228018

[B28] Petersen P. M. Hansen S. W. (1999). The course of long-term toxicity in patients treated with cisplatin-based chemotherapy for non-seminomatous germ-cell cancer. Ann. Oncol. 10, 1475–1483 10643539 10.1023/a:1008322909836

[B29] Reintsch W. E. Mandato C. A. McCrea P. D. Fagotto F. (2008). Inhibition of cell adhesion by xARVCF indicates a regulatory function at the plasma membrane. Dev. Dyn. 237, 2328–2341 10.1002/dvdy.21651 18729204

[B30] Ross C. J. Katzov-Eckert H. Dube M. P. Brooks B. Rassekh S. R. Barhdadi A. (2009). Genetic variants in TPMT and COMT are associated with hearing loss in children receiving cisplatin chemotherapy. Nat. Genet. 41, 1345–1349 10.1038/ng.478 19898482

[B31] Roth B. J. Greist A. Kubilis P. S. Williams S. D. Einhorn L. H. (1988). Cisplatin-based combination chemotherapy for disseminated germ cell tumors: long-term follow-up. J. Clin. Oncol. 6, 1239–1247 2457658 10.1200/JCO.1988.6.8.1239

[B32] Sinisi A. A. Pasquali D. Notaro A. Bellastella A. (2003). Sexual differentiation. J. Endocrinol. Invest. 26, 23–28 12834017

[B33] von Schlippe M. Fowler C. J. Harland S. J. (2001). Cisplatin neurotoxicity in the treatment of metastatic germ cell tumour: time course and prognosis. Br. J. Cancer 85, 823–826 10.1054/bjoc.2001.2006 11556831 PMC2375074

[B34] Wanderas E. H. Fossa S. D. Tretli S. (1997). Risk of subsequent non-germ cell cancer after treatment of germ cell cancer in 2006 Norwegian male patients. Eur. J. Cancer 33, 253–262 10.1016/S0959-8049(96)00458-3 9135497

[B35] Weinshilboum R. M. (2006). Pharmacogenomics: catechol O-methyltransferase to thiopurine S-methyltransferase. Cell. Mol. Neurobiol. 26, 539–561 10.1007/s10571-006-9095-z 16807786 PMC11520626

